# The Association Between Treatment Interval and Survival in Patients With Colon or Rectal Cancer: A Systematic Review

**DOI:** 10.1007/s00268-021-06188-z

**Published:** 2021-06-26

**Authors:** Ruud F. W. Franssen, Maud T. A. Strous, Bart C. Bongers, F. Jeroen Vogelaar, Maryska L. G. Janssen-Heijnen

**Affiliations:** 1 Department of Clinical Physical Therapy, VieCuri Medical Center, Venlo Tegelseweg, Venlo, 210 5912BL The Netherlands; 2 Department of Epidemiology, GROW School for Oncology and Developmental Biology, Faculty of Health, Medicine and Life Sciences, Maastricht University, Maastricht, The Netherlands; 3 Department of Surgery, VieCuri Medical Center, Venlo, The Netherlands; 4 Department of Nutrition and Movement Sciences, School of Nutrition and Translational Research in Metabolism (NUTRIM), Faculty of Health, Medicine and Life Sciences, Maastricht University, Maastricht, The Netherlands; 5 Department of Epidemiology, Care and Public Health Research Institute (CAPHRI), Faculty of Health, Medicine and Life Sciences, Maastricht University, Maastricht, The Netherlands; 6 Department of Epidemiology, VieCuri Medical Center, Venlo, The Netherlands

## Abstract

**Background:**

Surgery for colon or rectal cancer is associated with a high incidence of complications, especially in patients with a low aerobic fitness. Those patients might benefit from a comprehensive preoperative workup including prehabilitation. However, time between diagnosis and treatment is often limited due to current treatment guidelines. To date, it is unclear whether the treatment interval can be extended without compromising survival.

**Methods:**

A systematic review concerning the association between treatment intervals and survival in patients who underwent elective curative surgery for colon or rectal cancer was performed. A search up to December 2020 was conducted in PubMed, Cinahl and Embase. Original research articles were eligible. Quality assessment was performed using the Downs and Black checklist.

**Results:**

Eleven observational studies were included (897 947 patients). In colon cancer, treatment intervals that were statistically significant associated with reduced overall survival or cancer-specific survival ranged between > 30 and > 84 days. In rectal cancer, only one out of four studies showed that treatment intervals > 49 days was associated with reduced cancer-specific survival.

**Conclusions:**

This systematic review identified that studies investigating the association between treatment intervals and survival are heterogeneous with regard to treatment interval definitions, treatment interval time intervals and used outcome measures. These aspects need standardization before a reliable estimate of an optimal treatment interval can be made. In addition, further research should focus on establishing optimal treatment intervals in patients at high risk for postoperative complications, as particularly these patients might benefit from extended diagnosis to treatment intervals permitting comprehensive preoperative preparation.

**Supplementary Information:**

The online version contains supplementary material available at 10.1007/s00268-021-06188-z.

## Introduction

The main curative treatment of colon and rectal cancer is surgical resection of the tumor, with or without (neo-)adjuvant treatment. Despite advances in surgery and anesthesia, complication rates for the main curative treatment of colorectal cancer, being surgical tumor resection, remain high (20–50%) [1–3]. Postoperative complications are associated with a delayed or inadequate recovery of physical fitness levels after surgery [4], reduced survival [5] and earlier cancer recurrence [6].

The time between first clinical presentation and cancer treatment is a complex pathway separated by several milestones. The term diagnostic interval is used to refer to the period between first clinical presentation and diagnosis. Time between diagnosis and first treatment is called treatment interval. Although the length of both the diagnostic interval and the treatment interval might impact survival, especially the latter is relevant in relation to optimizing a patient’s physical fitness in anticipation of their cancer treatment [7].

Interventions aiming at optimizing a patient’s physical fitness (including aerobic fitness) before the start of treatment (e.g., surgery) are called prehabilitation [8]. Two recent studies have shown that 3 to 6 weeks of prehabilitation in anticipation of abdominal surgery can effectively improve preoperative aerobic fitness and reduce postoperative complications by ~ 50% [9, 10]. However, there is an inter-individual variation in the response to prehabilitation with regard to improvements in aerobic fitness, implying that some patients might benefit more from a longer program duration [10, 11].

Nevertheless, most societies have strict treatment interval time targets (34 days in the Netherlands [12]), that are not based on solid evidence [13], but leave a limited time window for a comprehensive preoperative workup. Extending the time interval between diagnosis and surgery could open a window for a comprehensive individualized and personalized prehabilitation program aiming at an optimal preparation of high-risk patients in anticipation of the upcoming stress of hospitalization and surgery.

Time between diagnosis and treatment seems trivial since the development of a colon or rectal adenocarcinoma may take 10 years or more [14]. However, with regard to the exponential growth of most malignancies, risk for metastasis could be the highest in these last few weeks [15, 16].

Although evidence is emerging, it remains unclear whether the treatment interval (TI) can be safely extended without compromising (cancer-free) survival. Therefore, the aim of this systematic review was to evaluate if, and to what extent, TI can be extended in patients with colon or rectal cancer scheduled for elective surgery, without compromising overall, cancer-specific or cancer-free survival.

## Material and methods

A systematic review of the literature was conducted according to the Preferred Reporting Items for Systematic Reviews and Meta-Analyses (PRISMA) guidelines [17]. The search string (Supplemental File A) that was executed in the databases PubMed, Embase and Cinahl up to December 2020, included patients diagnosed with colon or rectal cancer who underwent elective curative surgical treatment (population), length of TI or short versus longer TI (exposure and comparator) and overall survival, cancer-specific survival or cancer-free survival (outcome). No filters were applied. In addition, reference lists of included studies were checked for additional relevant studies. Definition of TI was extracted from the articles. Original studies that assess TI on a continuous scale as well as studies using TI intervals, with survival as an outcome, written in English, German or Dutch were eligible. Studies in which patients participated in an intervention prior to cancer treatment and studies only focusing on diagnostic delay were excluded. Due to the differences between colon and rectal cancer with respect to cancer recurrence, tumor biology and pathology, and cancer treatment [18], studies that did not present separate analyses for colon and rectal cancer were excluded.

Title and abstract of the retrieved records, and subsequently full text articles were screened for eligibility, independently by two researchers (RF and MS) using Rayyan QCRI [19]. In case of disagreement between the reviewers, a third reviewer (MJ) was consulted.

Quality assessment of the studies was performed by two reviewers (RF and MS) independently using the Downs and Black checklist for non-randomized studies [20]. The Downs and Black checklist consists of 27 questions regarding quality of reporting, internal and external validity, and power of the included studies. Data extraction was performed by the first author (RF) and verified on accuracy and completeness by the second author (MS).

## Results

**Fig. 1:**
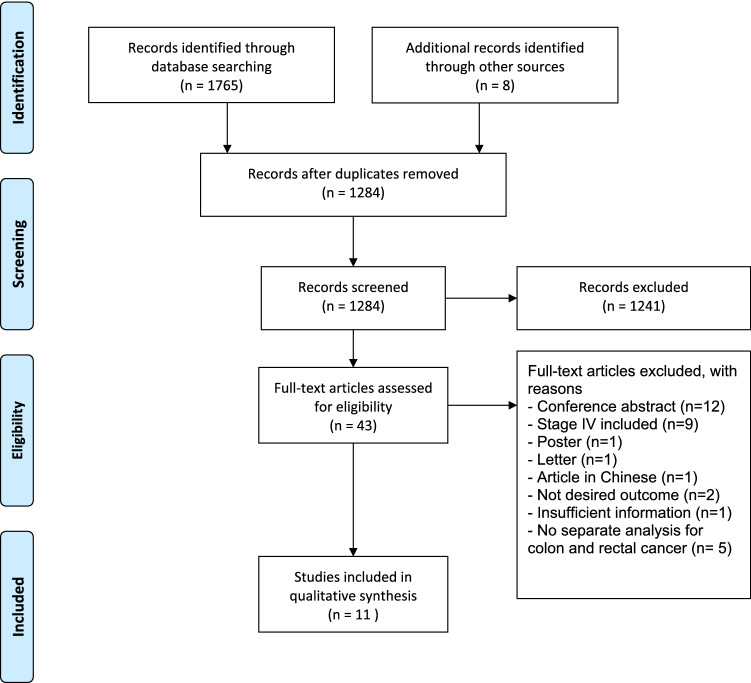
PRISMA flow diagram, displaying the selection of studies and reasons for exclusion

A total of 11 studies were included (see Fig. 1 for the PRISMA flowchart of included studies).

The included studies had a total sample size of 897 947 patients, ranging from 266 in the smallest study [21] to 514 103 in the largest study [22]. Studies originated from different geographical locations: seven studies from the USA [22–28], one from the UK [29], two from the Netherlands [30, 31] and one from Mexico [21]. Studies were published between 2010 and 2020. Study designs comprised database reviews (*n* = 5) [22, 26–29], retrospective (*n* = 2) [23, 30] and prospective (*n* = 3) [21, 24, 31] cohort studies, and a matched case–control study (*n* = 1) [25]. Ten studies [21–30] analyzed colon cancers and four analyzed rectal cancer [25, 29–31], of which one study also analyzed tumors of the recto-sigmoid as a separate entity [29].

The start of TI (diagnosis) was not described clearly in six studies [22, 26–29, 31]. In other studies, the definition of the time point used as diagnosis differed. One study used the date of the first investigation defining malignancy [24], while some studies used date of colonoscopy or first specialist consultation as the date of diagnosis [21, 23, 30]. Others used the date of confirmed pathological diagnosis as date of ‘diagnosis’ [25]. End of TI was defined as the date of surgery in nine studies [21–29]. In the two remaining studies, end of TI was defined as the date of start of the earliest cancer treatment (either surgery, neoadjuvant radiotherapy and/or chemotherapy) [30, 31].

Follow-up duration was not stated in four studies [22, 27–29]. In the remaining studies, median follow-up ranged from 2.4 to 5.4 years. Outcome in the majority of the studies (7 out of 10) was overall survival (OS) [21–24, 26, 27, 30], two studies reported on relative survival (RS) [29, 31], one study reported on all-cause death (ACD) and cancer-specific death (CSD) [25], and one study used cancer-specific mortality (CSM) [28]. Cancer-free survival (CFS) as an outcome was reported in four studies [24, 30, 31]. For readability of this manuscript, the term overall survival (OS) was used for the outcome measures OS and ACD. Cancer-specific survival (CSS) was used for RS, CSD and CSM, and CFS was used for the outcome measure CFS. A full overview of study characteristics of the included studies is presented in Table 1.

**Table 1:** Characteristics of included studies

Authors, year, country	Tumor site (stage)	Sample size (% female)	Age (years)	Study design, evidence level [32]	Start of, end of and median treatment interval	Analysis	Analysis adjusted for	Follow-up period
Bagaria et al. 2018 USA [23]	Colon (stage I–III)	4 685 (47.6%)	Mean ± SD 71 ± 11	Retrospective cohort, 2b	**Start:** Diagnosis by colonoscopy **End:** Surgery **Median:** 11 days (range 1–256)	Cox HR	Patient characteristics Comorbidity Tumor characteristics	Median, range 5.4, 1 day–24.6 years
Gleason et al. 2020 USA	Colon (stage I–III)	21 408 (56.6%)	*n* = 8 755 60–75 years *n* = 12 653 > 75 years	Database review, 2b	**Start:** Diagnosis, not specified **End:** Surgery **Median:** 25 days	Cox HR	Patient characteristics Comorbidity Tumor characteristics Treatment characteristics	Not specified
Grass et al. 2020 USA [26]	Colon (stage I–III)	118 504 (52%)	Median, IQR 69, 59–78	Database review, 2b	**Start:** Diagnosis, not specified **End:** Surgery **Median:** 24 days (IQR 16–36)	Cox HR	Socioeconomic state Hospital Year of diagnosis/treatment	Median, 5.3 years
Kaltenmeier et al. 2019 USA [22]	Colon (stage I–III)	514 103 (52.6%)	Median, IQR 72, 61–80	Database review, 2b	**Start:** Diagnosis, not specified **End**: Surgery **Median:** 14 Days (IQR 4–27)	Cox HR	Patient characteristics Comorbidity Tumor characteristics Socioeconomic state Hospital	Not specified
Kucejko el al 2020 USA [27]	Colon (stage I–III)	187 319 (53.3%)	Mean ± SD 68.5 ± 13.5	Database review, 2b	**Start:** Diagnosis, not specified **End:** Surgery **Median:** Not reported	Cox HR	Patient characteristics Tumor characteristics	Not specified
Lino Silva et al. 2019 Mexico [21]	Colon (stage I–III)	266 (51.1%)	Median, IQR 57, 47–68	Prospective cohort, 2b	**Start:** First specialist consultation **End:** Surgery **Median:** 38 days	Kaplan–Meier	Not applicable	Not specified
Wanis et al. 2017 USA [24]	Colon (stage I–III)	908 (50.2%)	*n* = 47 < 50 years *n* = 101 50–59 years *n* = 225 60–69 years *n* = 293 70–79 years *n* = 242 ≥ 80 years	Prospective cohort, 1b	**Start**: First investigation defining malignancy **End:** Surgery **Median:** 38 days (IQR 21–61)	Cox HR	Patient characteristics (for DFS only) Tumor characteristics Treatment characteristics	Median 2.7 years
Gort et al 2010 The Netherlands [31]	Rectum (stage I–III)	819 (38.2%) 755 ^b^	Median, range 68, 25–92	Prospective cohort, 1b	**Start:** Diagnosis, not specified **End:** Cancer treatment (radiotherapy, surgery) **Median:** 40 days (IQR 28–53)	Cox HR	Patient characteristics Comorbidity Tumor characteristics Treatment characteristics	Median, IQR 4.4, 3.2–5.6 years
Pruitt et al. 2013 USA [25]	Colon and rectal (local–regional–distant)^a^	Colon: 2 634 (55.7%) Controls: 4 064 (61.1%)	*n* = 287 66–69 years *n* = 508 70–74 years *n* = 618 75–79 years *n* = 631 80–84 years *n* = 590 ≥ 85 years	Matched case–control, 2b	Start: Confirmed pathologic diagnosis End: Surgery **Median:** Colon 13 days, Rectum 16 days	Weighted logistic regression	Patient characteristics Comorbidity Tumor characteristics Treatment characteristics Socioeconomic state Hospital (for ACD only) Year of diagnosis/treatment	Median 29.9 months
Redaniel et al. 2014 UK [29]	Colon, recto-sigmoid and rectal (Dukes stage A and B)	Colon: 29 431 Recto-sigmoid: 4 249 Rectum 12 831 Total: 46 511 (43.9%)	*n* = 921 15–44 years *n* = 2 744 24–54 years *n* = 8 628 55–64 years *n* = 15 507 65–74 years *n* = 18 711 ≥ 75 years	Database review, 2b	**Start:** Diagnosis by first event of highest priority, in declining priority: 1) histological or cytological confirmation; 2) admission to the hospital; 3) first consultation outpatient clinic **End:** Surgery **Median:** 30 days (IQR 18–42)	Cox eHR	Patient characteristics Tumor characteristics Socioeconomic state	Not specified
Strous et al. 2019 the Netherlands [30]	Colon and rectal (stage I–III)	Colon: 559 Rectum: 231 Total: 790 (45.7%)	Mean ± SD 70 ± 10	Retrospective cohort, 2b	**Start:** Date of biopsy **End:** Neoadjuvant treatment or Surgery **Median:** 32 days (IQR 26–43)	Cox HR	Patient characteristics Comorbidity Tumor characteristics Postoperative complications	Median, IQR 50, 32–75 months

*CI* confidence interval, *IQR* interquartile range, *SD* standard deviation, *UK* United Kingdom, *USA* United States of America

^a^Results displayed separately for disease stage

^b^Sample size for the CFS model is lower due to exclusion of patients who died within 30 days postoperatively, patients with metastasis within 3 months and patients without tumor-free margins

### Methodological quality

As all studies were observational studies, no study reached the maximum score of 28 on the Downs and Black quality checklist. Quality scores ranged between 16 and 22. The greatest differences were seen in the items concerning reporting, ranging from a lowest score of six [22, 27] to a highest score of ten [23, 30], as well as in the items about confounding, ranging from a score of two [21] to a score of four [29, 30] (see Table 2).

**Table 2:** Results of the quality assessment of the included studies according to the Downs and Black checklist

Author (year)	Reporting^a^	External validity^a^	Bias^a^	Confounding^a^	Power^a^	Total^b^
Bagaria et al. (2018) [23]	10	3	4	3	1	21
Gleason et sl. (2020)	7	3	4	3	1	18
Grass et al. (2020) [26]	7	3	4	3	1	18
Kaltenmeier el al. (2019) [22]	6	3	4	3	1	17
Kucejko et al. (2020) [27]	6	3	4	3	1	17
Lino Silva et al. (2019) [21]	7	3	4	2	1	17
Wanis et al. (2017) [24]	8	3	4	3	1	19
Gort et al. (2010) [31]	8	3	4	3	1	19
Pruitt et al. (2013) [25]	6	3	3	3	1	16
Redaniel et al. (2014) [29]	8	3	4	4	1	20
Strous et al. (2019) [30]	10	3	4	4	1	22

When the Downs and Black checklist referred to an intervention, this was conceived as exposed (a long time to treatment initiation) versus non-exposed (a short time to treatment initiation). Question 27 regarding power was scored on a binary scale: sufficient sample size (1) and insufficient sample size (0). Sample size was estimated based on the number of uncensored events in combination with the amount of predictor parameters that was corrected for in the survival analysis (one in ten rule)

^a^The maximal possible score for separate items of the Downs and Black checklist was: reporting 11; external validity 3; bias 7; confounding 6; power 1

^b^The total maximal possible score was 28

### Time to treatment initiation and survival in colon cancer

Associations between TI and OS or CSS in colon cancer were reported in ten studies [21–30], of which four studies [21, 24, 25, 30] found no association between TI and OS (Table 3). In contrast, six studies found a significant negative or a U-shaped association between TI and OS [22, 23, 26, 27] or CSS [28, 29]. Thresholds indicating that longer TI was associated with reduced OS or CSS ranged between > 30 and > 84 days.

**Table 3:** Associations between time to treatment initiation and survival in patients with colon cancer

Authors, year	Tumor stage	Associations of treatment intervals with survival		
Bagaria et al. 2018 [23]	I–III	*OS*		
		TI 1–7 days,		reference category
		TI 8–14 days,		HR of 1.02 (95% CI 0.92–1.14)
		TI 15–21 days,		HR of 1.03 (95% CI 0.90–1.17)
		TI 22–28 days,		HR of 1.05 (95% CI 0.89–1.23)
		TI 29–35 days,		HR of 1.12 (95% CI 0.92–1.36)
		TI 36–42 days,		HR of 1.14 (95% CI 0.89–1.46)
		TI 43–49 days,		HR of 1.11 (95% CI 0.79–1.56)
		TI 50–53 days,		HR of 1.17 (95% CI 0.89–1.60)
		TI 63–84 days,		HR of 1.07 (95% CI 0.73–1.57)
		TI > 84 days,		**HR of 1.47 (95% CI 1.02–2.11)**
Gleason et al. 2020 [28]	I–III	CSM		
			Patients 60–75 years old	Patients > 75 years old
		TI 0–10 days,	**HR of 1.98 (95% CI 1.64–2.41**	**HR of 1.91 (95% CI 1.70–2.16)**
		TI 11–20 days,	**HR of 1.65 (95% CI 1.36–2.00)**	**HR of 1.74 (95% CI 1.55–1.97)**
		TI 21–30 days,	**HR of 1.50 (95% CI 1.23–1.81)**	**HR of 1.52 (95% CI 1.35–1.72)**
		TI 31–40 days,	HR of 1.1.1 (95% CI 0.89–1.38)	HR of 1.02 (95% CI 0.88–1.17)
		TI 41–50 days,	reference category	
		TI 51–60 days,	**HR of 1.34 (95% CI 1.04–1.71)**	HR of 1.08 (95% CI 0.91–1.28)
		TI 61–70 days,	HR of 1.03 (95% CI 0.75–1.42)	**HR of 1.37 (95% CI 1.13–1.66)**
		TI 71–80 days,	HR of 1.29 (95% CI 0.87–1.91)	**HR of 1.44 (95% CI 1.13–1.84)**
		TI 81–90 days,	HR of 1.49 (95% CI 0.93–2.37)	HR of 1.29 (95% CI 0.93–1.78)
		TI > 90 days	**HR of 1.23 (95% CI 1.06–1.42)**	**HR of 1.72 (95% CI 1.44–2.06)**
Grass et al. 2020 [26]	I–III	*OS* **HR of 1.06 (95% CI 1.05–1.07)** HR represents increase in risk for every 14 days of extra TI > 40 days (as a continuous variable)		
Kaltenmeier et al. 2019 [22]	I–III	*OS*		
		TI < 7 days,		**HR of 1.56 (95% CI 1.45–1.68)**
		TI 7–30 days,		reference category
		TI 31–60 days,		**HR of 1.13 (95% CI 1.02–1.25)**
		TI 61–90 days,		**HR of 1.49 (95% CI 1.19–1.85)**
		TI 91–120 days,		**HR of 2.28 (95%CI 1.61–3.23)**
		TI 121–180 days,		**HR of 2.46 (95% CI 1.48––4.09)**
Kucejko el al 2020 [27]	I–III	*OS*^*a*^		
		Patients up to 65 years old		Patients > 65 years old
		TI ≤ 14 days,	**HR of 1.38 (95% CI 1.32–1.44)**	**HR of 1.42 (95% CI 1.39–1.46)**
		TI 15–28 days,	reference category	
		TI 29–42 days,	HR of 0.99 (95% CI 0.92–1.05)	HR of 1.02 (95% CI 0.99–1.06)
		TI 43–84 days,	**HR of 1.22 (95% CI 1.13–1.31)**	**HR of 1.12 (95% CI 1.12–1.21)**
		TI > 84 days,	**HR of 1.68 (95% CI 1.46–1.93)**	**HR of 1.35 (95% CI 1.26–1.44)**
Lino Silva et al. 2019 [21]	I–III	*OS* Stage I: Not determined due to small sample size Stage II: TI 0–24 days TI 25–38 days TI 39–60 days TI > 60 days Log-rank p = 0.829 Stage III: TI 0–24 days TI 25–38 days TI 39–60 days TI > 60 days Log-rank p = 0.936		
Wanis et al. 2017 [24]	I–III	*OS*		
		TI ≤ 30 days,		reference category
		TI 31–60 days,		HR of 0.91 (95% CI 0.66–1.26)
		TI 61–90 days,		HR of 0.82 (95% CI 0.53–1.26)
		TI 91–120 days,		HR of 0.78 (95% CI 0.34–1.81)
		TI > 120 days,		HR of 0.90 (95% CI 0.48–1.70)
		*CFS*		
		TI ≤ 30 days,		reference category
		TI 31–60 days,		HR of 0.84 (95% CI 0.55–1.29)
		TI 61–90 days,		HR of 0.95 (95% CI 0.58–1.61)
		TI 91–120 days,		HR of 1.46 (95% CI 0.58–3.72)
		TI > 120 days,		HR of 0.48 (95% CI 0.15–1.53)
Pruitt et al. 2013 [25]	local–regional–distant^a^	*ACD* Local stage:		
		TI < 7 days,		**adjusted OR of 1.43 (95% CI 1.04–1.96)**
		TI 7–14 days,		reference category
		TI 14–28 days,		adjusted OR of 1.18 (95% CI 0.86–1.62)
		TI ≥ 28 days,		adjusted OR of 1.15 (95% CI 0.80–1.64)
		Regional stage:		
		TI < 7 days,		adjusted OR of 1.24 (95% CI 0.94–1.63)
		TI 7–14 days,		reference category
		TI 14–28 days,		adjusted OR of 1.13 (95% CI 0.85–1.49)
		TI ≥ 28 days,		adjusted OR of 1.06 (95% CI 0.75–1.50)
		*CSD*		
		Local stage:		
		TI < 7 days,		adjusted OR of 1.14 (95% CI 0.69–1.89)
		TI 7–14 days,		reference category
		TI 14–28 days,		adjusted OR of 0.84 (95% CI 0.51–1.38)
		TI ≥ 28 days,		adjusted OR of 0.71 (95% CI 0.40–1.25)
		Regional stage:		
		TI < 7 days,		adjusted OR of 1.26 (95% CI 0.95–1.67)
		TI 7–14 days,		reference category
		TI 14–28 days,		adjusted OR of 1.04 (95% CI 0.78–1.40)
		TI ≥ 28 days,		adjusted OR of 0.78 (95% CI 0.54–1.14)
Redaniel et al. 2014 [29]	Dukes stage A and B	*RS*		
		TI < 25 days,		**excess HR of 1.71 (95% CI 1.50–1.94)**
		TI 25–38 days,		reference category
		TI > 38 days,		**excess HR of 1.19 (95% CI 1.02–1.38)**
Strous et al. 2019 [30]	I–III	*OS*		
		TI ≤ 35 days,		reference category
		TI > 35 days,		HR of 1.29 (95% CI 0.90–1.86)
		*CFS*		
		TI ≤ 35 days,		reference category
		TI > 35 days,		HR of 1.21 (95% CI 0.78–1.90)

*ACD* all-cause death, *CFS* cancer-free survival, *CI* confidence interval, *CSD* cancer-specific death, *DFS* disease-free survival, *eHR* excess hazard ratio, *HR* hazard ratio, *IQR* interquartile range, *OR* odds ratio, *OS* overall survival, *RER* relative excess risk, *RS* relative survival, *SD* standard deviation, *TI* treatment interval, *UK*  United Kingdom, *USA* United States of America

^a^The original study displayed results of two databases, results of the Medicare database are not displayed as also non-elective surgery was included. Presented data is from the NCDB database

CFS was reported as an outcome measure in two studies [24, 30]. No significant associations between TI and CFS was found, with a TI up to > 120 days (Table 3).

### Time to treatment initiation and survival in rectal cancer

In rectal cancer, three out of four studies did not find an association between TI and OS [25, 30] or CSS [25, 29]. One study [31] showed that patients with stage I–III rectal cancer who started treatment (surgery or neoadjuvant radiotherapy) > 49 days after diagnosis had reduced CSS (Table 4).

**Table 4:** Associations between time to treatment initiation and survival in patients with rectal cancer

Authors, year,	Tumor stage	Associations of treatment intervals with survival	
Gort et al. 2010 [31]	I–III	*RS*	
		TI ≤ 49 days,	reference category
		TI > 49 days,	**RER of 1.51 (95% CI 1.01–2.27)**
		*CFS*	
		TI ≤ 49 days,	reference category
		TI > 49 days**,**	**HR of 1.44 (95% CI 1.06–1.96)**
Pruitt et al. 2013 [25]	Local–regional–distant^a^	*ACD*	
		Local stage:	
		TI < 7 days,	adjusted OR of 1.50 (95% CI 0.90–2.51)
		TI 7–14 days,	reference category
		TI 14–28 days,	adjusted OR of 1.49 (95% CI 0.93–2.40)
		TI ≥ 28 days,	adjusted OR of 1.45 (95% CI 0.88–2.40)
		Regional stage:	
		TI < 7 days,	adjusted OR of 1.11 (95% CI 0.70–1.76)
		TI 7–14 days,	reference category
		TI 14–28 days,	adjusted OR of 0.79 (95% CI 0.51–1.22)
		TI ≥ 28 days,	adjusted OR of 1.05 (95% CI 0.65–1.70)
		*CSD*	
		Local stage:	
		TI < 7 days,	adjusted OR of 1.55 (95% CI 0.77–3.10)
		TI 7–14 days,	reference category
		TI 14–28 days,	adjusted OR of 1.52 (95% CI 0.80–2.92)
		TI ≥ 28 days,	adjusted OR of 1.63 (95% CI 0.83–3.18)
		Regional stage:	
		TI < 7 days,	adjusted OR of 1.02 (95% CI 0.65–1.58)
		TI 7–14 days,	reference category
		TI 14–28 days,	adjusted OR of 0.83 (95% CI 0.54–1.26)
		TI ≥ 28 days,	adjusted OR of 0.74 (95% CI 0.46–1.19)
Redaniel et al. 2014 [29]	Dukes stage A and B	*RS*	
		Recto-sigmoid:	
		TI < 25 days,	excess HR of 1.31 (95% CI 0.96–1.79)
		TI 25–38 days,	reference category
		TI > 38 days,	excess HR of 1.03 (95% CI 0.74–1.45)
		Rectum:	
		TI < 25 days,	excess HR of 1.17 (95% CI 0.97–1.39)
		TI 25–38 days,	reference category
		TI > 38 days,	excess HR of 1.11 (95% CI 0.94–1.32)
Strous et al. 2019 [30]	I–III	*OS*	
		TI ≤ 35 days,	reference category
		TI > 35 days,	HR of 0.86 (95% CI 0.46–1.61)
		*CFS*	
		TI ≤ 35 days,	reference category
		TI > 35 days,	HR of 1.21 (95% CI 0.65–2.25)

*ACD* all-cause death, *CFS* cancer-free survival, *CI* confidence interval, *CSD* cancer-specific death, *DFS* disease-free survival, *eHR* excess hazard ratio, *HR* hazard ratio, *IQR* interquartile range, *OR* odds ratio, *OS* overall survival, *RER* relative excess risk, *RS* relative survival, *SD* standard deviation, *TI* treatment interval, *UK* United Kingdom, *USA* United States of America

With regard to CFS, one study [30] did not show a significant association between a TI of > 35 days and CFS whereas another study [31] showed that a TI > 49 days was associated with shorter CFS.

### Discussion

This systematic review aims to evaluate to what extent the TI can be safely extended without compromising survival in patients with colon or rectal cancer in order to identify a safe time frame for prehabilitation. In colon cancer, six out of ten studies showed a significant association between a longer TI and reduced OS or CSS. Of these, one study found an association with an excessively long TI of > 84 days [23] and five studies with a TI ranging between > 30 and > 51 days [22, 26, 27, 29]. No associations were found between TI and CFS in patients with colon cancer [24, 30].

In rectal cancer, only one [31] out of four studies showed that patients had a better OS and CFS when treated (surgery or radiotherapy) within 49 days of diagnosis.

The associations between TI and OS or CSS in colon cancer are in contrast with a review investigating the effect of time from diagnosis to surgery on oncological outcomes in patients with colon cancer [14]. In this systematic review by Hangaard Hansen et al. [14], no associations were found between longer delays and reduced survival. Although their review was published in 2018, the current review managed to identify seven new studies that were not previously reviewed systematically. In addition, Hangaard Hansen et al. [14] also included patients with stage IV colon cancer. The inclusion of patients with stage IV disease might have attenuated a possible association, as these patients have markedly lower survival rate compared to patients with stage I–III colon cancer [33]. Regarding rectal cancer, the current review is the first to collectively examine studies investigating the association between TI and survival in rectal cancer as a unique entity.

Although some studies show that longer delays seem to be associated with reduced survival, there is no consensus on the length of the TI from which this association becomes significant. This inconsistency might be partially explained by the variety in time points that were considered as diagnosis, and therefore as starting point of the TI. Duration of the TI might vary significantly between different starting points, such as date of biopsy or diagnosis by confirmed pathology. However, the lack of a consistent starting point of the TI does not fully explain the broad range of 31 to 84 days that is observed in colon cancer. The variety in findings does however identify a major pitfall in the current literature. Studies included in this systematic review were heterogeneous regarding their methodology, definition of TI, definition of TI time intervals and used outcome measures (such as OS, RS, ACD, CFS, CSM). Therefore, comparison of studies is difficult and an optimal or maximal TI is difficult to establish. All of these key aspects need standardization before reliable estimates can be made regarding the association between TI and survival in patients with colon or rectal cancer. Another limitation of the current review was that only a part of the interval between presentation of symptoms and first treatment was studied. Although the TI is of main interest with regard to the aim of this review, the association between TI and survival might be biased by the length of the diagnostic interval.

In colon cancer, four studies [22, 26, 27, 29] reporting reduced survival with longer TIs were large retrospective database studies (combined sample size of 866 437 patients). These database studies did not adjust for some relevant confounders such as comorbidity, adjuvant treatment and postoperative complications. Previous studies have shown that postoperative complications are related to both survival [5, 6, 30], cancer recurrence [6] and inadequate recovery of physical fitness postoperatively [4]. Also, three out of these four studies [22, 26, 27] used the same database that was complete for only 70% of newly diagnosed cancer cases. Although the latter must introduce some bias, it is impossible to determine how it exactly affects the results.

Some studies (*n* = 4) showed that a very short TI (e.g., shorter than one week) was associated with reduced survival [22, 25, 34]. Although most studies explicitly stated that emergency surgery was excluded from the analyses, a very short TI probably represents patients with intestinal obstructions that were not designated as emergency surgery but still had higher priority. Previous research showed that patients with intestinal obstructions form a subgroup of patients with a short TI that also have a poorer prognosis [35]. In addition, one study found that a short TI of < 30 days was associated with reduced CSS [28]. However, the association was lower when a complete preoperative workup, including endoscopy, CT scan of the pelvis and abdomen, and carcinoembryonic antigen, was performed. This indicates that the increased risk associated with a short TI, might be mitigated by a full preoperative oncologic workup. The authors concluded that ideal timing for surgery was between 3 and 6 weeks after diagnosis allowing time for the clinician to complete preoperative workup and for the patient to prepare for surgery and organize their social support network.

Perhaps, more emphasis could be given to how the TI can be used optimally in association with complications and survival, instead of focusing on a short TI. A study that did not observe an association between TI and OS, did contrastingly find a significant association between OS and variables associated with frailty, such as a higher age and postoperative complications[30] in colon cancer, and age and comorbidities[30] in rectal cancer. Although more research is needed, this could mean that the effect size of these risk factors is higher, and therefore probably more instrumental than a short TI. This is also emphasized in the study of Redaniel et al. [29], who indicated that factors associated with frailty, such as a higher age and deprivation state, were associated with RS in patients with CRC independent of TI.

Prehabilitation aims to increase a patient’s health between diagnosis and surgery in order to reduce postoperative complications and enhance recovery postoperatively [8]. In high-risk patients with colon or rectal cancer, there could be trade-off between the medical urgency to operate on and creating sufficient time preoperatively for an optimal preparation for surgery. Although not specifically aiming at high-risk patients, a recent Canadian study indeed showed that prehabilitation improved CFS in patients with colon and rectal cancer [36].

Studies aiming at identifying a safe window for prehabilitation, should give more emphasis to the association between TI and CFS, as it is a much more sensitive variable than OS given the relatively high 5-year survival rates in colon and rectal cancer. Only a few studies (*n* = 3) investigated the association between CFS and TI [24, 30, 31]. In patient with colon cancer no association were observed between TI and earlier cancer recurrence whereas in patients with rectal cancer TI up to 49 days did not lead to reduced CFS. On the other hand, especially in elderly patients, OS might also be important, as elderly have increased odds of dying from other causes than cancer recurrence.

Future research could be improved by using a uniform definition for the start and end of the TI. In addition, length of the TI time intervals should be standardized in order to increase comparability between studies. With regard to the maximal time frame for prehabilitation, the start of the TI should ideally be set to the first investigation defining malignancy (such as endoscopy, computed tomography scan), as this is the first possible starting point for prehabilitation. In addition, perhaps multiple starting points can be reported to increase comparability between studies. Furthermore, studies should adjust for important confounders, such as postoperative complications, comorbidities and adjuvant treatment in addition to age, sex and tumor stage. Lastly, the association between TIs and (cancer-free) survival should be specifically investigated in patients who have a high risk (based on low preoperative aerobic fitness) for postoperative complications, as these patients might benefit most from a comprehensive preoperative workup.

## Conclusion

Studies are heterogeneous with regard to treatment interval definitions, treatment interval time intervals and used outcome measures. *These key aspects need standardization before a reliable estimate can be made regarding an optimal TI.* Previous trials have shown that prehabilitation with a program duration of 3–6 weeks, can effectively reduce postoperative complications. However, individual patients might benefit more from a more extensive time window. There is an urgent need for high-quality studies in large cohorts, in which colon and rectal cancer are studied separately with uniformly defined TI start and time intervals. Moreover, subgroup analyses for patients with a high risk for postoperative complications are needed in order to further clarify the association between TI and (cancer-free) survival in this subgroup of patients who are expected to benefit the most from a comprehensive preoperative prehabilitation program.

## Supplementary Information

Below is the link to the electronic supplementary material.

Supplementary file1 (DOCX 12 kb)
