# Supplementary material for: The Association Between Treatment Interval and Survival in Patients With Colon or Rectal Cancer: A Systematic Review
Source: World J Surg. 2021 Jun 26;45(9):2924–37. doi: 10.1007/s00268-021-06188-z (PMC8322003; doi:10.1007/s00268-021-06188-z)
Supplement: Supplementary file 1 — Supplementary file1 (DOCX 12 kb) [file 268_2021_6188_MOESM1_ESM.docx]

Supplemental File A. PubMed search strategy.

| P |
| --- |
| "colorectal-neoplasms"[MeSH] OR colorectal-neoplasm[tiab] OR colorectal-neoplasms[tiab] OR colorectal-neoplasia[tiab] OR colorectal-tumor[tiab] OR colorectal-tumors[tiab] OR colorectal-tumour[tiab] OR colorectal-tumours[tiab] OR colorectal-carcinoma[tiab] OR colorectal-carcinomas[tiab] OR colorectal-cancer[tiab] OR colorectal-cancers[tiab] OR colorectal-malignancy[tiab] OR colorectal-malignancies [tiab] OR colon-neoplasm[tiab] OR colon-neoplasms[tiab] OR colon-neoplasia[tiab] OR colon-tumor[tiab] OR colon-tumors[tiab] OR colon-tumour[tiab] OR colon-tumours[tiab] OR colon-carcinoma[tiab] OR colon-carcinomas[tiab] OR colon-cancer[tiab] OR colon-cancers[tiab] OR colon-malignancy[tiab] OR colon-malignancies [tiab] OR colonic-neoplasm[tiab] OR colonic-neoplasms[tiab] OR colonic-neoplasia[tiab] OR colonic-tumor[tiab] OR colonic-tumors[tiab] OR colonic-tumour[tiab] OR colonic-tumours[tiab] OR colonic-carcinoma[tiab] OR colonic-carcinomas[tiab] OR colonic-cancer[tiab] OR colonic-cancers[tiab] OR colonic-malignancy[tiab] OR colonic-malignancies [tiab] OR rectal-neoplasm[tiab] OR rectal-neoplasms[tiab] OR rectal-neoplasia[tiab] OR rectal-tumor[tiab] OR rectal-tumors[tiab] OR rectal-tumour[tiab] OR rectal-tumours[tiab] OR rectal-carcinoma[tiab] OR rectal-carcinomas[tiab] OR rectal-cancer[tiab] OR rectal-cancers[tiab] OR rectal-malignancy[tiab] OR rectal-malignancies [tiab] OR cancer-of-the-colon[tiab] OR cancer-of-the-rectum[tiab] |
| E |
| "time-to-treatment"[MeSH] OR time-to-treatment[tiab] OR time-to-treatments[tiab] OR waiting-time[tiab] OR waiting-times[tiab] OR door-to-treatment-time[tiab] OR therapeutic-delay[tiab] OR therapeutic-delays[tiab] OR waiting-period[tiab] OR waiting-periods[tiab] OR waiting-time[tiab] OR waiting-times[tiab] OR wait-time[tiab] OR wait-times[tiab] OR wait-period[tiab] OR wait-periods[tiab] OR provider-delay[tiab] OR provider-delays[tiab] OR surgery-delay[tiab] OR surgery-delays[tiab] OR time-to-surgery [tiab] OR delayed-treatment[tiab] OR delayed-treatments[tiab] OR treatment-delay[tiab] OR treatment-delays[tiab] OR optimal-timing[tiab] |
| O |
| "survival"[MeSH] OR survival[tiab] OR "mortality"[MeSH] OR mortality[tiab] OR death-rate[tiab] OR death-rates[tiab] OR progression[tiab] OR recurrence[tiab] OR Time-to-failure[tiab] OR "prognosis"[MeSH] OR prognosis[tiab] OR prognoses[tiab] OR "morbidity"[MeSH] OR morbidity[tiab] OR morbidities[tiab] |
